# Necrotizing ANCA-Positive Glomerulonephritis Secondary to Culture-Negative Endocarditis

**DOI:** 10.1155/2015/649763

**Published:** 2015-12-27

**Authors:** Sophie Van Haare Heijmeijer, Dunja Wilmes, Selda Aydin, Caroline Clerckx, Laura Labriola

**Affiliations:** ^1^Department of Nephrology, Clinique Saint-Pierre, Ottignies, Belgium; ^2^Department of Internal Medicine, Cliniques universitaires Saint-Luc, Université catholique de Louvain, Brussels, Belgium; ^3^Department of Pathology, Cliniques universitaires Saint-Luc, Université catholique de Louvain, Brussels, Belgium; ^4^Department of Nephrology, Cliniques universitaires Saint-Luc, Université catholique de Louvain, Brussels, Belgium

## Abstract

Infective endocarditis (IE) and small-vessel vasculitis may have similar clinical features, including glomerulonephritis. Furthermore the association between IE and ANCA positivity is well documented, making differential diagnosis between IE- and ANCA-associated vasculitis particularly difficult, especially in case of culture-negative IE. We report on one patient with glomerulonephritis secondary to culture-negative IE caused by* Bartonella henselae *which illustrates this diagnostic difficulty.

## 1. Introduction

Infective endocarditis (IE) and small-vessel vasculitis may have a similar clinical presentation. Cardiac involvement has been documented in patients with Wegener granulomatosis [1]. Conversely, patients with IE often have renal complications, including glomerulonephritis. Moreover the association between IE and ANCA positivity is well documented, making differential diagnosis between IE- and ANCA-associated vasculitis particularly difficult, especially in case of culture-negative IE. We report on one patient with glomerulonephritis secondary to culture-negative IE caused by* Bartonella henselae* which highlights this diagnostic difficulty.

## 2. Case

A 67-year-old man presented with asthenia and weight loss (12 kg over 5 months). His medical history included hypertension, bicuspid aortic valve, and thoracic aortic aneurysm repair. He owned four pet dogs and one cat. Medications included amlodipine and acetylsalicylic acid. There was no history of recent dental procedure or use of intravenous drugs. He did not report fever, chills, night sweats, hematuria, or oliguria. On admission he was afebrile and normotensive. Physical examination revealed palpable purpura on his legs without edema.

Three months prior to admission, serum creatinine and CRP levels were 1.6 mg/dL [*e*GFR 43 mL/min/1.73 m^2^ by the 4-variable MDRD (Modification of Diet in Renal Disease Study equation)] and 2.1 mg/dL, respectively. On admission laboratory data revealed serum creatinine 3.5 mg/dL (*e*GFR 18 mL/min/1.73 m^2^), serum urea 88 mg/dL, hemoglobin 8.8 g/dL, normal platelet count, and moderate inflammatory syndrome (CRP 2.3 mg/dL) with polyclonal hypergammaglobulinemia. Urinalysis showed hematuria (1370 red blood cells/*μ*L) with red blood cell casts and moderate proteinuria (protein/creatinine ratio 0.72 g/g; 830 mg/24 h). Further laboratory investigations revealed both positive myeloperoxidase antineutrophil cytoplasmic (MPO-ANCA) (89 RU/mL, normal <20) and proteinase 3 antibodies (PR3-ANCA) (41 RU/mL, normal <20); low complement levels of C3 (80 mg/dL; normal 90–180 mg/dL); normal C4 levels; and increased rheumatoid factor (189 IU/mL; normal <40). Anti-nuclear and anti-cardiolipin antibodies were negative. There was no cryoglobulinemia. Serologic tests for hepatitis B, C and HIV were negative. Multiple blood cultures were negative.

**Figure 1:**
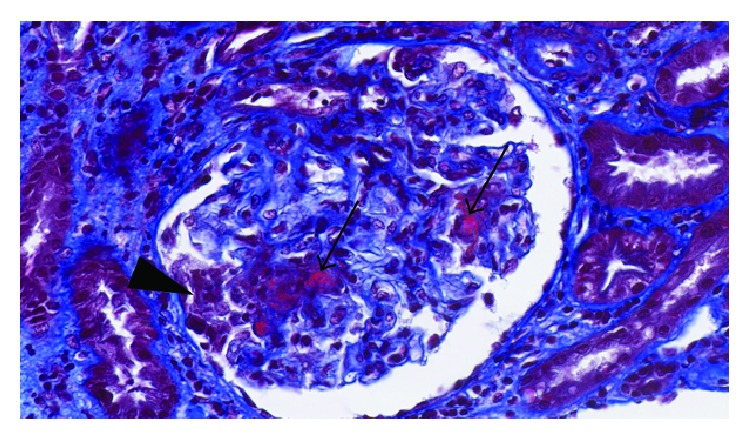
Kidney biopsy findings (Masson's trichrome, 46x): glomeruli showing foci of necrosis (arrows) with a recent cellular crescent (arrowhead).

**Figure 2:**
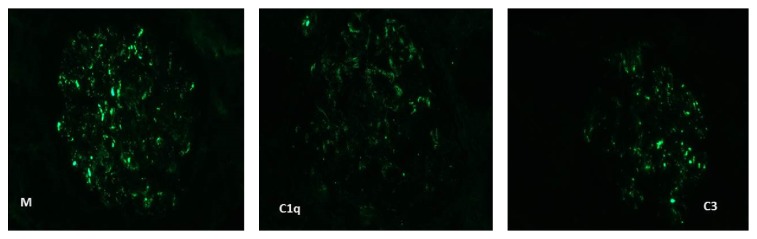
Immunofluorescence shows subepithelial granular staining for IgM and C3 and parietal granular staining for C1q.

Renal biopsy showed segmental necrosis involving 1 out of 16 nonsclerotic glomeruli, as well as one cellular crescent and one fibrous crescent (Figure 1). Two glomeruli were globally sclerosed. Foci of acute interstitial infiltrate and acute tubular necrosis were associated. Mild significant interstitial fibrosis was observed. Immunofluorescence showed moderate granular parietal staining for IgM, C3, and C1q (Figure 2), which suggested infection-related glomerulonephritis. However transesophageal echocardiography did not show any signs of endocarditis. Imaging by positron-emission tomography only showed mild splenomegaly.

Because of this rapidly progressive renal failure secondary to necrotizing and crescentic glomerulonephritis without any evidence of infective endocarditis, treatment with cyclophosphamide (500 mg) and intravenous pulses of methylprednisolone was started, followed by oral corticotherapy (1 mg/kg/day). The patient felt rapidly better and renal function improved.

However, ten days later, creatinine level acutely peaked at 9.2 mg/dL and hemodialysis was started. Two weeks later the patient presented with thoracic pain. Coronary angiography showed a double coronary vessel disease. Repeated transesophageal echocardiography showed severe aortic and moderate mitral insufficiency with possible perforation. Broad-spectrum antibiotics were started and the patient underwent mitral valve repair, aortic valve replacement, and triple coronary artery bypass. He had an uneventful postoperative course. Hemodialysis therapy was discontinued 13 days later. Histopathological examination of the excised valve was consistent with bacterial endocarditis, but cultures of the valve remained negative. Serological test results for atypical pathogens turned negative except for* Bartonella henselae* (IgM: 1/100; IgG: 1/1280).* Bartonella henselae* DNA was detected by polymerase chain reaction (PCR) on the resected valve. The patient was administered doxycycline and gentamycin for two weeks. He was discharged home and continued treatment by doxycycline and rifampicin for four more weeks. Methylprednisolone doses were slowly tapered. Three months later, creatinine level was 1.5 mg/dL, and ANCA were negative.

## 3. Discussion

Renal disease in the setting of IE is a well-known extracardiac complication of IE, affecting as many as 40–50% of patients with IE [2]. Kidney lesions include abscess from septic emboli, immune complex-mediated glomerulonephritis, ANCA-associated glomerulonephritis, and renal toxicity secondary to antibiotics.

Among the causes of infection-related glomerulonephritis in adults, 6–20% are related to endocarditis [3] and glomerulonephritis is considered as a minor Duke criterion for the diagnosis of IE [4]. In a recent large biopsy-based clinicopathologic series on IE-related glomerulonephritis (*n* = 49), acute kidney injury was the most common presenting condition (79%) with hematuria present in almost all cases. However, typical nephritic and nephrotic syndromes were relatively uncommon (9 and 6%, resp.) [5]. Hypocomplementemia (low C3 and/or C4 levels) was found in 56% of patients tested and ANCA antibodies in as many as 28%, with mostly anti-PR3 specificity, although anti-MPO and dual-positive anti-PR3 and anti-MPO were also seen. As in previous reports (summarized in [3]), the most frequent infectious organism identified on blood cultures was* Staphylococcus aureus* (53%), followed by* Streptococcus* species (23%) [5]. Importantly, culture-negative endocarditis accounts for almost 10% of the patients with IE-related glomerulonephritis [5, 6].

The spectrum of glomerular histopathologic lesions of IE-related glomerulonephritis is variable. Diffuse necrotizing crescentic GN is the most common pattern (53%), followed by diffuse proliferative glomerulonephritis (37%) and mild mesangial hypercellularity without endocapillary proliferation or crescents (10%) [5]. The immunofluorescence patterns include classic postinfectious type (with prominent IgG and C3 deposition and subepithelial humps on electron microscopy), although IgM- or IgA-dominant staining (particularly with* Staphylococcus aureus*) has also been reported with variable frequency [3, 5, 7]. Interestingly, the association between IE and pauci-immune glomerulonephritis has been documented in as many as 44% of cases [5].

Our patient has experienced blood culture negative endocarditis, which represents 2.5–31% of all cases of IE [8]. The most often, previous antibiotic therapy is the reason for negative blood cultures. The namely “true” blood culture-negative IE is caused by intracellular bacteria that cannot be routinely cultured in blood with currently available techniques. In a large reference laboratory series,* Bartonella *sp. was the second most common aetiology in Europe (28%), after* Coxiella burnetii *[8].* B. quintana* has been associated with body-louse infested alcoholic homeless persons, whereas* B. henselae* has been associated with patients with exposure to cats or their fleas, and previous structural valvulopathy, with most cases occurring in native aortic valves [8].

Diagnostic tests for* Bartonella* IE include cultures and serologic and DNA amplification techniques. PCR from valvular tissue has been demonstrated to have a higher sensitivity than blood and valvular culture (92% versus 20 and 31%, resp.), even in patients receiving antibiotics [8]. In a recent large series of* Bartonella* IE, serum Western blotting (WB), PCR, and 16S rRNA gene amplification in valvular tissue exhibited sensitivities of 100%, 92%, and 60%, respectively [17]. Although PCR on total blood and serum may enable the diagnosis before cardiac surgery, its sensitivity is low (33% and 36%, resp.) [17].* Bartonella* species IgG titer >1 : 800 using an immunofluorescence assay has a predictive value of 95% to detect* Bartonella* IE, but a titer <800 does not exclude the diagnosis [17, 18]. Therefore, any patient with IgG titer <800 and a medical history evocative of IE should be tested by WB and PCR following cardiac valve removal. Currently, it is considered that a positive PCR from a cardiac valve or blood specimen, an IgG titer of >800, and a positive WB assay should be major criteria for* Bartonella* IE [17].

Glomerular disease in the setting of* Bartonella* IE seems to be a rare complication, but its true incidence is unknown. To the best of our knowledge, there have been only 10 cases published to date (Table 1); all of them are caused by* B. henselae*. Hypocomplementemia was documented in the 6 patients in whom complement levels were tested. ANCA were positive in 7/11 cases, with anti-PR3 specificity in 6. Of note, dual anti-PR3 and anti-MPO specificity was documented in only one case (this present case). The predominant light microscopy pattern of injury was segmental necrotizing crescentic glomerulonephritis. On immunofluorescence, the predominant immunoglobulin was IgM, with strong C3 deposits, although in two cases there was a dominant staining for IgA [11] and IgG [14]. Only two cases showed a pauci-immune pattern. Most cases required valvular surgery. Interestingly, in 7/11 cases, immunosuppressive agents were first administered for a suspected vasculitis and withdrawn when the diagnosis of endocarditis was made.

This case highlights the difficulty to make the differential diagnosis between occult infection with secondary glomerular involvement and ANCA-associated vasculitis. Distinguishing these two conditions is crucial, particularly when immunosuppressive treatment is considered. Although delayed, the diagnosis of culture-negative IE was finally suggested by a proliferative immune complex-mediated glomerulonephritis in a patient with a structural cardiac valve anomaly. The association of rapidly progressive renal failure secondary to necrotizing and crescentic glomerulonephritis and palpable purpura on the legs without any evidence of IE has initially supported the diagnosis of ANCA-associated vasculitis. However, even in case of negative blood cultures, the concomitant presence of hypocomplementemia and positive ANCA antibodies is strongly suggestive of immune complex-mediated disease. Moreover the dual-positive ANCA specificity anti-PR3 and anti-MPO is very unusual and should always trigger the screening for secondary causes of ANCA positivity, that is, occult infection, including IE due to atypical pathogens [5], or levamisole-contaminated cocaine use [19].

## Figures and Tables

**Table 1:** Characteristics of published cases of *Bartonella* endocarditis-associated glomerulonephritis.

Case	Serum complement level		ANCA	Light microscopy	IF microscopy	Diagnostic	Therapy
	C3	C4					
van Tooren et al. [9]	ND	ND	Negative	Diffuse proliferative GN Focal crescents	ND	Serology	Antibiotics Valvular surgery

Bookman et al. [10] Case 1	↓	↓	Negative	Segmental necrosis Crescents	IgM-dominant staining C3 deposits	Serology	Prednisolone Antibiotics

Bookman et al. [10] Case 2	N	↓	Negative	Segmental necrosis Crescents	IgM-dominant staining C3 deposits	Serology	Prednisolone Antibiotics Valvular surgery

Bookman et al. [10] Case 3	↓	↓	Negative	Segmental necrosis Crescents	IgM-dominant staining C3 deposits	Serology	Antibiotics Valvular surgery

Turner et al. [11]	ND	ND	Anti-PR3	Focal crescents	IgA-dominant staining	PCR on valvular tissue	Prednisolone Cyclophosphamide Antibiotics Valvular surgery

Vikram et al. [12]	ND	ND	Anti-PR3	Segmental necrosis Focal crescents	Pauci-immune	Serology PCR on valvular tissue	Prednisolone Cyclophosphamide Antibiotics Valvular surgery

Forbes et al. [13]	↓	↓	Anti-PR3	Diffuse proliferative GN	IgG/C3/C1q	Serology	Antibiotics

Salvado et al. [14]	↓	N	Anti-PR3	Diffuse proliferative GN Diffuse crescents	IgM/C3/C1q	Serology	Antibiotics

Khalighi et al. [15]	↓	↓	Anti-PR3	Diffuse proliferative GN Focal crescents	IgM-dominant staining C3/C1q deposits	Serology PCR on blood	Antibiotics Prednisolone

Shah et al. [16]	ND	ND	Anti-PR3	Necrotizing GN	Pauci-immune	Serology Culture of valvular tissue	Prednisolone Cyclophosphamide Antibiotics Valvular surgery

Present report	↓	N	Anti-PR3 and anti-MPO	Focal/segmental necrosis/crescents	IgM/C3/C1q	Serology PCR on valvular tissue	Prednisolone Cyclophosphamide Antibiotics Valvular surgery

Ref. IF: immunofluorescence; GN: glomerulonephritis; N: normal; and ND: not determined.
